# Labor force participation during COVID-19 and risk of depression: a Danish register study

**DOI:** 10.1093/eurpub/ckac168

**Published:** 2022-11-18

**Authors:** Sophie S Hellmann, Sanne P Møller, Annette K Ersbøll, Ziggi I Santini, Maj Britt D Nielsen, Morten K Grønbæk, Ola Ekholm, Lau C Thygesen

**Affiliations:** National Institute of Public Health, University of Southern Denmark, Copenhagen, Denmark; National Institute of Public Health, University of Southern Denmark, Copenhagen, Denmark; National Institute of Public Health, University of Southern Denmark, Copenhagen, Denmark; National Institute of Public Health, University of Southern Denmark, Copenhagen, Denmark; National Institute of Public Health, University of Southern Denmark, Copenhagen, Denmark; National Institute of Public Health, University of Southern Denmark, Copenhagen, Denmark; National Institute of Public Health, University of Southern Denmark, Copenhagen, Denmark; National Institute of Public Health, University of Southern Denmark, Copenhagen, Denmark

## Abstract

**Background:**

COVID-19 caused economic insecurity for businesses and their employees. Understanding effects of changes in labor force participation on depression risk during economic recession is fundamental for early diagnosis. The study evaluates if changes in labor force participation are associated with depression risk during COVID-19 in Denmark.

**Methods:**

A register-based longitudinal study of Danes aged 25–67 years without depression 2 years prior to baseline defined as February 2020. An eight-level categorical variable on stable or changing labor force participation was defined from monthly employment percentage gradients in the Danish Register-based Evaluation and Marginalization Database from February 2020. The cohort was followed until 31 December 2020 for depressions overall and mild-, moderate- and severe depression. Sex-stratified cox regression models with hazard ratios (HR) and 95% confidence intervals (95% CI) were performed accounting for important confounders.

**Results:**

In total, 1 619 240 (50.3%) men of mean age 45.6 years and 1 598 587 (49.7%) women of mean age 45.9 years were included. Becoming unemployed implied an increased HR of depression in men (HR 2.02; 95% CI 1.94–2.10) and women (2.19; 2.12–2.26) compared to a steady-state full-time employment. Being outside the labor force or employed part-time implied an elevated HR in men (3.02; 2.82–3.23 and 2.41; 2.35–2.48) and women (3.13; 2.30–3.31 and 2.30; 2.26–2.35), respectively, compared to a steady-state full-time employment.

**Conclusions:**

Changes in labor force participation were associated with higher risk of depression relative to a steady-state full-time employment particularly among individuals with low labor force participation during COVID-19.

## Introduction

Labor force participation is an important contributor to mental well-being as an important social identity marker.^1^^,^^2^ Involuntary loss of employment is associated with compromised mental well-being, increased risk of psychiatric diagnoses and hazardous health behavior.^3–5^ A substantial body of research has found a higher risk of depression in unemployed and among individuals with non-standard employment, and evidence has shown that the fear of becoming unemployed could be an even stronger predictor of depression and other mental health disorders than being unemployed.^6^ Employment status is a strong predictor for socioeconomic status, which in turn is known to be strongly positively related to mental well-being.^7^

From past pandemics and the financial crisis in 2008, evidence has shown an association between lack of labor force participation and mental disorders, such as stress, depression, anxiety and suicidal behavior with a rise in demand for psychotropic drugs.^8–10^ With the onset of the COVID-19 pandemic (COVID-19) and the subsequent societal lockdown by governments worldwide, many businesses were forced to reorganize and reduce labor costs to remain afloat either by actual dismissals, cuts in employment hours, higher demand for labor force flexibility or other non-standard employment.

A longitudinal Danish study with self-reported data on mental well-being measured before and during COVID-19 has recently reported a significant decrease in mental well-being most predominant in otherwise healthy and higher educated individuals.^11^ This association is supported by other longitudinal studies primarily measuring the early COVID-19 effects on mental health finding an increase in incidence of depression.^12^^,^^13^

Labor force participation is a predictor for self-reported depressive symptoms in several cross-sectional studies^14–17^ and longitudinal studies^14^^,^^15^^,^^18–25^ confirming lack of labor force participation to be a risk factor for self-reported depressive symptoms during COVID-19. Studying changes in labor force participation during COVID-19 could add to current evidence reflecting the impact of external stressors on risk of depressive disease among employed. Such evidence is addressed by some longitudinal studies^14^^,^^15^^,^^18–25^ affirming predominantly a positive association but in perspective of a few longitudinal self-reported measures on labor force participation and mental health disease. The current large register-based longitudinal cohort study explores the relationship between changes in labor force participation during COVID-19 and clinically diagnosed depressions measuring a change in effects from objective monthly longitudinal measures on labor force participation and clinical depression diagnoses.

## Methods

### Population and setting

The study was conducted as a nationwide register-based longitudinal cohort study. We included the Danish population with permanent residence in Denmark obtained from the Danish Civil Registration Register.^26^ The population was age-restricted to 25–67 years of age at baseline to approximate homogeneity in the adult labor force. The retirement age is currently 66.5 years in Denmark, dynamic, and modified by industry and political regulation.

We included Danish residents without diagnosed depression within 2 years prior to baseline on the 28 February 2020, just before the first national lockdown in Denmark. The official Danish lockdown during the first wave of COVID-19 was initiated by the Danish Government on 11 March 2020 with closing of country boarders, schools, daycares, universities, non-essential businesses, cultural institutions and an official governmental regulation for increased remote work, social distancing and quarantine protocols. A gradual first phase reopening was initiated on 13 April 2020 followed by a Phase 2 reopening from 7 May 2020–27 June 2020 with partial opening of country borders for low COVID-19 incidence countries. A rise in the COVID-19 incidence rate occurred during the fall and winter of 2020 with a partial reinstallation of some of the restrictions from the first wave of COVID-19. Economically, Denmark experienced a decrease in gross national product by 4% in 2020 and a temporary increase in unemployment, which returned to the level before the COVID-19 pandemic around the second half of 2020.

### Exposure

We obtained information on labor force participation from the Danish Register-based Evaluation and Marginalization Database (DREAM) until 31 December 2020 providing monthly information on the personal employment percentage gradient.^27^ We included the employment percentage gradient as a measure of employment status. The employment percentage gradient is digitally processed from all monthly salary payments by Danish employers to the Danish tax authorities and DREAM. We categorized the employment percentage gradient to actual employment hours per week (full-time: ≥37; part-time: 30–36; part-time: <30).

The exposure variable was defined in eight categories as stable or changing labor force participation: staying employed full-time, staying employed part-time 30–36, staying employed part-time <30, employed increased employment hours, employed decreased employment hours, unemployed becoming employed, employed becoming unemployed and staying outside the labor force for heterogenous reasons of receipt of social benefits, such as labor force disabilities, sick leave, maternity leave, education or retirement. The change was established as a change from baseline just before COVID-19 defined as February 2020 until (i) April 2020 as the short-term change and (ii) August 2020 as the long-term change.

### Outcome

The primary outcome was depression during follow-up until 31 December 2020. Information on depression was obtained from the primary and secondary healthcare sector in Denmark. Clinically diagnosed depression in the primary healthcare sector was obtained from the Danish National Prescription Registry. The Danish National Prescription Registry pertains individual-level redemption of antidepressants on Danish community pharmacies. We defined depression as having at least two redemptions of antidepressants as classified according to the Anatomical Therapeutic Chemical Classification System (ATC) by the ATC code: NO6A. Clinically diagnosed depression in the secondary healthcare sector was obtained from the Danish National Patient Register including in- and outpatients contacts to somatic and psychiatric hospitals.^28^ All clinical depression diagnoses were classified according to the ICD-10 codes for depression: F32 and F33.

A sensitivity analysis was furthermore performed where depression was divided into ‘mild’: F32.0, F32.1, F32.8, F32.9, F32.9A, F33.0, F33.00, F33.01, F34.11, ‘moderate’: F32.10, F32.11, F33, F33.4, F33.8, F33.9, F33.10, F33.11, F34.10, F34.11 and ‘severe’: F32.2, F32.3, F32.30, F32.31, F33.2, F33.3, F33.30, F33.31. This analysis could only be performed for individuals with hospital contacts.

### Covariates

Charlson comorbidity index 10-years before baseline was furthermore categorized from the Danish National Patient Register until February 2020.^29^ Highest attained educational level was obtained from the Danish Population Education Register.^30^ Information on industry was obtained from the DREAM database.

The *a priori* defined confounders were included in the analyses as categorical covariates: sex (male and female), age by quintile distribution (25–32, 33–42, 43–49, 50–57 and 58–67), ethnicity (originating from Western or non-Western countries), marital status (married, unmarried, divorced and widowed), area of residence (capital, metropolitan, province, upland and country municipalities), education (basic school, upper secondary or vocational, bachelor level and higher education), Charlson comorbidity index (0: none; 1–2: moderate; and 3+: severe) and industry (16-level industry classification within areas of hospitality, culture, health, education, retail, manufacturing and transportation).

### Analysis

Descriptive statistics include mean (SD) and median (range) for continuous variables and proportions (%) for categorical variables. Cox regression analyses were conducted to estimate the association between a change in labor force participation and risk of depression by a calculation of Hazard ratios (HR) with 95% confidence intervals (95% CI) from (i) 1 May 2020 (short-term), and (ii) 1 September 2020 (long-term) as timely determined by the exposure variables and the mortality date or end of follow-up on 31 December 2020 (whichever came first) as right-censored variables. Semi-adjusted (age) and multivariate-adjusted (all covariates) models were used. The main analysis was stratified by sex. An analysis stratified by comorbidity was furthermore performed to explore a possible effect-modification. We furthermore explored by sensitivity analyses the association for disease severity in mild, moderate and severe depression among in-hospital contacts.

The study is entirely a register study, which according to Danish law and ethical guidelines needs no approval from an ethical committee. Analyses were conducted in SAS 9.4. All data were processed in the data secure remote server environment of Statistic Denmark.

## Results

Of 3 217 827 included individuals, 1 619 240 (50.3%) were men and 1 598 587 (49.7%) were women of mean age 45.6 and 45.9 years, respectively (table 1). The majority of men (88.8%) and women (88.4%) had Western origin. Half of the population in both sexes were married, but a higher proportion of women were widowed (2.1%) and had a higher educational level (40.9%) as compared to men. A similar proportion of men (13.1%) and women (13.9%) had comorbidity. Even though full-time employment was the most common labor force participation in both men (49.4%) and women (37.1%), a higher proportion of women (30.9%) than men (22.5%) held part-time employment and were employed in human health and social work (21.8%), whereas men (29.6%) were more likely employed in the manufacturing, construction and retail industry.

**Table 1:** Baseline characteristics of 3 217 827 Danes aged 25–67 years by sex

Characteristics	Men	Women
Total, *n* (%)	1 619 240 (50.3)	1 598 587 (49.7)
Age, years		
Mean (SD)	45.6 (12.3)	45.9 (12.3)
25–32	326 563 (20.2)	313 380 (19.6)
33–42	311 784 (19.2)	303 479 (19.0)
43–49	310 060 (19.1)	308 776 (19.3)
50–57	329 858 (20.4)	325 035 (20.3)
58–67	340 975 (21.1)	347 917 (21.8)
Ethnicity		
Western	1 437 345 (88.8)	1 413 829 (88.4)
Non-Western	166 698 (10.3)	172 339 (10.8)
Unknown	15 197 (0.9)	12 419 (0,8)
Marital status		
Married/partnership	782 490 (48.3)	828 006 (51.8)
Unmarried	641 057 (39.6)	506 791 (31.7)
Divorced	183 493 (11.3)	229 643 (14.4)
Widowed	12 200 (0.8)	34 147 (2.1)
Area of residence		
Capital	465 527 (28.8)	469 234 (29.3)
Metropolitan municipality	214 022 (13.2)	208 971 (13.1)
Province municipality	363 776 (22.5)	357 649 (22.4)
Upland municipality	254 818 (15.7)	251 653 (15.7)
Country municipality	320 049 (19.8)	310 266 (19.4)
Unknown	1048 (0.1)	814 (0.1)
Education		
Basic school	311 952 (19.3)	261 274 (16.3)
High school or vocational	816 654 (50.4)	660 423 (41.3)
Higher education	458 279 (28.3)	654 229 (40.9)
Unknown	32 355 (2.0)	22 661 (1.5)
Comorbidity		
0	1 407 531 (86.9)	1 376 405 (86.1)
1–2	177 865 (11.0)	194 228 (12.2)
≥3	33 844 (2.1)	27 954 (1.7)
Industry		
Agriculture, forestry and fishing	19 268 (1.2)	7694 (0.5)
Manufacturing	190 839 (11.7)	76 545 (4.8)
Construction	121 696 (7.5)	14 211 (0.9)
Wholesale and retail	168 065 (10.4)	113 073 (7.1)
Transportation and storage	81 216 (5.0)	22 880 (1.4)
Accommodation and food service	29 521 (1.8)	27 487 (1.7)
Information and communication	65 685 (4.1)	28 649 (1.8)
Financial and insurance	39 944 (2.5)	34 374 (2.2)
Real estate	19 227 (1.2)	12 605 (0.8)
Professional, scientific and technical	76 756 (4.7)	56 739 (3.5)
Administrative and support service	60 493 (3.7)	59 567 (3.7)
Public administration and defense	70 443 (4.4)	99 027 (6.2)
Education	73 705 (4.6)	119 862 (7.5)
Human health and social work	74 646 (4.6)	348 200 (21.8)
Arts, entertainment and recreation	17 812 (1.1)	17 729 (1.1)
Other service activities	38 746 (2.4)	34 576 (2.1)
Outside labor force and unknown	471 178 (29.1)	525 369 (32.9)
Labor force participation		
Employment hours per week		
Part-time <30	169 614 (10.5)	222 414 (13.9)
Part-time 30–36	194 449 (12.0)	271 768 (17.0)
Full-time ≥37	800 082 (49.4)	592 521 (37.1)
Outside labor force^a^	455 095 (28.1)	511 884 (32.0)

a Being outside labor force for various reasons: sickness leave, retirement, education or unknown.

More than half of the population did not experience any changes in their labor force participation during COVID-19 since 56.0% of men and 55.6% of women did not experience any changes in their labor force participation in the short-term perspective of COVID-19 (from February to April 2020) (table 2). Contrary, 17.5% of men and 14.1% of women did experience a short-term change in their labor force participation. Unemployment did increase during COVID-19 with 5.6% of men and women becoming unemployed from February to April 2020, which was not entirely counterbalanced by the 3.3% of men and women becoming employed in the same period (table 2). A large proportion of the study population remained out of the labor force in the same period with 26.4% of men and 30.3% of women. The same pattern was observed for the long-term period (from February to August 2020) although with higher proportions changing labor force participation and therefore lower proportions in stable labor force participation (Supplementary table S1). Women (63.4%) accounted for the majority of clinical diagnosed depressions (table 2).

**Table 2:** Sex-stratified associations of short-term changes (February 2020–April 2020) in labor force participation during COVID-19 with onset of depression in 3 216 194 Danes aged 25–67 years including clinical diagnoses from the primary and secondary healthcare sector in Denmark until 31 December 2020

Hazard ratio (HR)						
95% confidence interval (95% CI)	Total (%)	Cases	Person years	Incidence rate	HR (95% CI)^a^	HR (95% CI)^b^
**Men**	***N* = 1 618 249 (100)**	***N* = 89 910**	***N* = 1 059 968**	**Per 1000 person years**		

Employment hours per week						
No change						
<30	100 885 (6.2)	7856	65 451	120	2.77 (2.70–2.85)	2.41 (2.35–2.48)
30–36	96 862 (6.0)	3886	64 020	61	1.34 (1.30–1.39)	1.30 (1.26–1.35)
≥37	709 363 (43.8)	21 774	470 588	46	1.00	1.00
Increased employment hours	78 721 (4.9)	2827	52 153	54	1.23 (1.19–1.28)	1.21 (1.16–1.26)
Decreased employment hours	132 410 (8.2)	4772	87 720	54	1.21 (1.18–1.25)	1.21 (1.17–1.25)
Becoming employed	26 433 (1.6)	1429	17 348	82	1.94 (1.83–2.04)	1.75 (1.61–1.90)
Becoming unemployed	45 678 (2.8)	2718	29 897	91	2.14 (2.05–2.22)	2.02 (1.94–2.10)
Outside labor force^c^	427 897 (26.4)	44 648	272 791	164	3.63 (3.57–3.69)	3.02 (2.82–3.23)

**Women**	***N* = 1 597 945 (100)**	***N* = 155 748**	***N* = 1 029 604**	**Per 1000 person years**	**HR (95% CI)^a^**	**HR (95% CI)^b^**

Employment hours per week						
No change						
<30	150 919 (9.4)	17 863	96 554	185	2.52 (2.47–2.57)	2.30 (2.26–2.35)
30–36	206 351 (12.9)	15 511	134 818	115	1.48 (1.45–1.51)	1.37 (1.34–1.40)
≥37	531 470 (33.3)	26 988	350 146	77	1.00	1.00
Increased employment hours	70 082 (4.4)	4562	45 996	99	1.36 (1.32–1.41)	1.29 (1.25–1.34)
Decreased employment hours	83 390 (5.2)	5182	54 727	95	1.29 (1.25–1.33)	1.23 (1.20–1.27)
Becoming employed	27 614 (1.7)	2343	17 938	131	1.98 (1.89–2.06)	1.82 (1.70–1.94)
Becoming unemployed	44 326 (2.8)	4559	28 521	160	2.33 (2.25–2.40)	2.19 (2.12–2.26)
Outside labor force^c^	483 793 (30.3)	78 740	300 904	262	3.60 (3.55–3.65)	3.13 (2.30–3.31)

a Age-adjusted.

b Multivariate-adjusted: age, comorbidity, ethnicity, residence of living, marital status, education and industry.

c Being outside labor force for various reasons: sickness leave, retirement, education or unknown.

From the sex-stratified analysis we found that any change in labor force participation during COVID-19 was associated with a higher risk of depression as compared to staying in a steady-state full-time employment. Becoming unemployed during COVID-19 was particularly associated with higher risk of depression in men (HR 2.02; 95% CI: 1.94–2.10) and women (2.19; 2.12–2.26) in a short-term perspective during COVID-19 (table 2).

A lower degree of labor force participation than steady-state full-time employment during COVID-19 was consistently associated with an increased short-term risk of depression independent of sex (table 2). The multivariate-adjusted HR of depression was highest in men (3.02; 2.82–3.23 and 2.41; 2.35–2.48) and women (3.13, 2.30–3.31 and 2.30; 2.26–2.35) being outside the labor force or employed part-time hours, respectively, as compared to those staying in a steady-state full-time employment in the short-term perspective of COVID-19 (table 2).

The relationship of change in labor force participation with depression risk was not modified by comorbidity since a similarly higher multivariate-adjusted HR of depression persisted independently of level of Charlson comorbidity index during the short-term perspective of COVID-19 (table 3).

**Table 3:** Associations of short-term changes (February 2020–April 2020) in labor force participation during COVID-19 with onset of depression stratified by comorbidity in 3 216 194 Danes aged 25–67 years including clinical diagnoses from the primary and secondary healthcare sector in Denmark until 31 December 2020

Charlson comorbidity index	Comorbidity					
	Score 0		Scores 1–2		Score 3+	
Hazard ratio (HR)						
95% confidence interval (95% CI)	HR (95% CI)^a^	HR (95% CI)^b^	HR (95% CI)^a^	HR (95% CI)^b^	HR (95% CI)^a^	HR (95% CI)^b^
*N*/*E*=participants/events	*N* = 2 783 504		*N* = 371 511		*N* = 61 179	
	*E* = 186 614		*E* = 47 971		*E* = 11 073	
Employment hours per week						
No change						
<30	2.78 (2.73–2.83)	2.33 (2.29–2.37)	2.68 (2.58–2.79)	2.34 (2.25–2.43)	2.36 (2.14–2.60)	2.15 (1.94–2.37)
30–36	1.67 (1.64–1.70)	1.35 (1.32–1.37)	1.48 (1.42–1.55)	1.25 (1.20–1.31)	1.25 (1.09–1.44)	1.09 (0.94–1.25)
≥37	1.00	1.00	1.00	1.00	1.00	1.00
Increased employment hours	1.34 (1.31–1.38)	1.26 (1.22–1.29)	1.26 (1.18–1.36)	1.22 (1.14–1.31)	1.13 (0.92–1.40)	1.12 (0.91–1.39)
Decreased employment hours	1.23 (1.20–1.26)	1.22 (1.19–1.25)	1.17 (1.10–1.24)	1.18 (1.11–1.25)	0.97 (0.81–1.17)	1.01 (0.84–1.21)
Becoming employed	1.98 (1.91–2.05)	1.75 (1.66–1.86)	2.00 (1.83–2.18)	1.94 (1.69–2.22)	2.09 (1.68–2.59)	2.08 (1.48–2.91)
Becoming unemployed	2.24 (2.17–2.30)	2.09 (2.03–2.15)	2.41 (2.27–2.56)	2.28 (2.15–2.43)	1.95 (1.67–2.27)	1.91 (1.64–2.23)
Outside labor force^c^	3.53 (3.49–3.57)	3.00 (2.86–3.14)	3.76 (3.66–3.86)	3.41 (3.06–3.81)	3.63 (3.38–3.90)	3.38 (2.57–4.45)

a Age adjusted.

b Multivariate-adjusted: age, sex, ethnicity, residence of living, marital status, education and industry.

c Being outside labor force for various reasons: sickness leave, retirement, education or unknown.

The above associations were confirmed in a sensitivity analysis restricting the analysis to in-hospital diagnoses of clinical depression where the increased risk of clinical depression with weakened labor force participation persisted in mild, moderately and severely depressed (table 4). The stratified analysis for disease severity showed an amplified increased short-term multivariate-adjusted HR of depression among the moderately and severely depressed becoming unemployed (3.59; 3.22–4.01 and 3.01; 2.46–3.67) and staying outside the labor force (6.10; 4.92–7.57 and 3.72; 2.59–5.34) as compared to staying in a steady-state full-time employment (table 4). The results for a long-term change in labor force participation measured from February 2000 to August 2000 during COVID-19 showed similar associations (Supplementary tables S1–S3).

**Table 4:** Multivariate-adjusted associations of short-term changes (February 2020–April 2020) in labor force participation during COVID-19 with onset of mild, moderate and severe depression in 3 216 194 Danes aged 25–67 years including in-hospital clinical depression diagnoses until 31 December 2020

Clinical depression	Mild		Moderate		Severe	
	Events = 2176		Events = 10 911		Events = 3252	
Hazard ratio (HR)						
95% confidence interval (95% CI)	HR^a^	HR^b^	HR^a^	HR^b^	HR^a^	HR^b^
Employment hours per week						
No change						
<30	2.26 (1.86–2.75)	1.76 (1.44–2.15)	2.86 (2.63–3.10)	2.42 (2.22–2.64)	2.22 (1.91–2.59)	1.93 (1.65–2.25)
30–36	1.66 (1.36–2.03)	1.43 (1.16–1.77)	1.69 (1.55–1.86)	1.40 (1.28–1.55)	1.54 (1.31–1.80)	1.32 (1.12–1.56)
≥37	1.00	1.00	1.00	1.00	1.00	1.00
Increased employment hours	1.50 (1.15–1.97)	1.37 (1.04–1.80)	1.52 (1.34–1.72)	1.43 (1.26–1.62)	1.15 (0.91–1.46)	1.12 (0.88–1.42)
Decreased employment hours	1.20 (0.93–1.55)	1.14 (0.88–1.48)	1.60 (1.44–1.78)	1.62 (1.46–1.81)	1.14 (0.93–1.40)	1.21 (0.98–1.48)
Becoming employed	2.46 (1.75–3.46)	1.47 (0.86–2.51)	2.67 (2.29–3.10)	2.59 (2.01–3.33)	2.72 (2.10–3.52)	2.06 (1.34–3.16)
Becoming unemployed	3.80 (3.01–4.79)	3.05 (2.40–3.88)	3.85 (3.46–4.28)	3.59 (3.22–4.01)	3.30 (2.72–4.00)	3.01 (2.46–3.67)
Outside labor force^c^	6.07 (5.38–6.85)	3.03 (1.95–4.71)	6.56 (6.20–6.93)	6.10 (4.92–7.57)	5.23 (4.76–5.76)	3.72 (2.59–5.34)

a Age-adjusted.

b Multivariate-adjusted: sex, age, comorbidity, ethnicity, residence of living, marital status, education and industry.

c Being outside labor force for various reasons: sickness leave, retirement, education or unknown.

## Discussion

In this study, we explored the longitudinal association of changes in labor force participation during COVID-19 with onset of clinical depression in the Danish active labor force without a clinically diagnosed depression 2 years prior to COVID-19. We found that any change in labor force participation is associated with higher risk of depression in both sexes relative to a steady-state full-time employment during COVID-19. This association was particularly pronounced among individuals becoming unemployed or working reduced employment hours during COVID-19. The general higher risk of depression among those experiencing a change in their labor force participation during COVID-19 could be related to an unforeseen employment and economic insecurity known to induce mental illness in otherwise healthy individuals.^13^^,^^17^

Previous longitudinal studies^14^^,^^15^^,^^18–25^ are heterogenous and mainly based on self-reported web survey questionnaires studying short-term occupational effects of the initial phase of COVID-19 on depressive symptoms and mental distress. Except for one study,^25^ these longitudinal studies found an overall higher risk of reported mental health problems and depression among unemployed and part-time employed compared to full-time employed during COVID-19. Chandola et al.^19^ in the UK household longitudinal cohort study 2020 using data from repeated surveys comparing employment status before and after COVID-19 by a seven-category employment scale also reported unemployment and reduced employment hours to be the strongest predictors for common mental disorders among 13 754 British adults.

COVID-19 and the societal lockdown has caused economic unsafety and reduced mental well-being with increasing burden of mental distress, anxiety, financial worries, loneliness, abusive behavior and debut of mental illness in otherwise healthy individuals.^13^^,^^17^^,^^31^ COVID-19 has had severe economic consequences for entire industries particularly within fields of hospitality (restaurants, travel and tourism), culture, retail, manufacturing and transportation and has thus severely impacted public economic safety.^32^

Evidence has shown that labor force participation is an important predictor for mental health and that employment insecurity and unexpected disturbances in labor force participation from the negative economic effects of COVID-19 induce mental illness in otherwise healthy individuals.^13^^,^^17^ Previous Danish studies have also reported negative effects from the COVID-19 on mental health in the Danish population.^11^^,^^33^^,^^34^ From a recent Danish study by Thygesen et al.,^11^ the decrease in self-reported mental well-being was primarily found in otherwise healthy adults, whereas the self-reported well-being was less affected in those with low-mental well-being or clinically diagnosed depression before the COVID-19 pandemic.

The crude measures of depression from self-reported scales provided by current evidence makes it difficult to distinguish effects of changes in labor force participation during COVID-19 on actual clinical depressions from other mood disorders or a temporary state of being dispirited. Our study confirmed an association between the degree of labor force participation with risk of clinical depression, observing the highest risk of depression in those outside the labor force or with weakened labor force participation by reduced employment hours, and among employed experiencing unemployment during COVID-19.

Few longitudinal studies^21^^,^^24^^,^^25^ accounted for self-reported comorbidity finding significant positive associations with risk of mental health disease. Our study explored the relationship between changes in labor force participation with risk of clinical depression by somatic comorbidity, finding no effect-modification. This was in part contrasted by the results reported by Iob et al.^21^ from a prospective longitudinal cohort study in 2020 on 51 417 adults finding a significantly higher risk of both moderate and severe depression among individuals self-reporting having a physical health condition. This study used self-reported comorbidity and did not report on effect-modification by comorbidity within an association of change in employment status with risk of depression which could, at least in part, explain the differing results to this study.

The Danish labor market is based on a flexicurity system defined by a high level of financial support in the event of unforeseen unemployment or reduced labor force capability. COVID-19 had serious recessive effects on the Danish labor market causing marked decreases in revenues for 66% of the Danish companies.^32^ The Danish Government therefore introduced a salary relief package on 14 March 2020 encompassing temporary wage compensation structurally organized as a one-time transfer payment to the employers conditional on job security for the employees.^32^ The governmental employment subsidy package was evaluated to have had strong labor retaining effect in Denmark.^32^ An availability of societal welfare structures, such as salary aid programs, are generally closely associated with public health outcomes as socioeconomic determinants are important public health predictors.^35^ Advanced societal welfare structures generally provide a high level of economic and social support reducing the feeling of control-loss from sudden unemployment.^34^ Despite the finely developed welfare structures in Denmark, an elevated risk of depression from changes in employment during COVID-19 was found particularly in individuals becoming unemployed or holding part-time employment. These effects could be related to social marginalization, lack of meaningful structure on the day, loss of social status and the distressful feeling of lack of personal control.^36^

Mental health disabilities can cause unemployment and thus reverse causality among individuals *a priori* at higher risk of unemployment from their mental illness or underlying poorer general health status compared to those staying employed.^6^ Specifically, we addressed this possibility by excluding individuals with a depression diagnosis within 2 years before baseline. The change in occupational status during the COVID-19 lockdown as an external variable could thereby be considered being closer to causal inference of occupational status. Solid evidence exists that effects of unemployment on mental health is mediated by effects of loss of income and thus financial strains.^35^ Evidence consistently supports a causal association of low socioeconomic position with depression onset and duration,^37^ being likely also an underlying explanation for the effects found in our study of an increased depression risk particularly among unemployed and part-time employed having low income status as compared to the steady-state full-time employed.^38^

An important strength of our study is the large nationwide and longitudinal data on labor force participation and clinically diagnosed depressions. Demographic, labor force and mental disease information was obtained from nationwide complete register linkage of very high internal and external validity minimizing risk of selection bias. Psychiatric health care and hospitalization is free of charge in Denmark with no private psychiatric in-patient facilities. The large data gave high power in our analyses and the ability to explore the influence of changes in employment status during COVID-19. This could contribute with valuable insight into possible ‘at higher risk populations’ from the mental health consequences caused by sudden changes in employment during COVID-19. The clinical registry data enabled accounting for pre-existing depressive disease and somatic comorbidity, which is a further valuable strength of this study.

Individuals with psychiatric diseases are treated in both the primary- and secondary healthcare sector in Denmark. Even though we had access to registry information on in-hospital psychiatric treatment of individuals with depressive episodes, most of depressive episodes in Denmark are diagnosed and treated in the primary healthcare sector by general practitioners or psychiatrists. To account for this possible limitation, we included individual-level purchase of psychotropic medication from the Danish Pharmaceutical Register to ensure depression diagnosis from a treatment duration of at least one repetitive buy of the prescribed psychotropic drug.

Our study pertained only to effects of COVID-19 in year 2020 and did not account for further time-varying effects of changes in labor force participation with depression risk. However, evidence has suggested that acute loss of employment might be stronger associated with depression risk than periods of longer unemployment, possibly explained by mechanisms of psychological adaption, resilience and better general coping strategies over time.^39^ This hypothesis needs further testing in data with a longer follow-up period.

We did not account for possible seasonal changes in the distribution of depressions, which is a known predictor for depression of substantial higher proportion during wintertime in Denmark.^40^ Since this study mainly provides insights into a relatively short-term effect of unemployment on depression risk, we find that seasonal fluctuations or increasing incidence rates of depression over time are unlikely to explain the results of this study.

Evidence from the financial crisis in 2008 has suggested that a manifestation of mental disorders from stressful periods induced by societal economic hardships, employment insecurity and unemployment might have a long incubation time with higher prevalence in the post-crisis period where the social acceptability of unemployment might be lower than during the actual economic crisis possibly due to changes in social norms.^10^ Further research on long-term effects on mental health from the COVID-19 is needed to address mental health risk distribution in vulnerable subpopulations who might suffer from marginalization. These long-term mental health effects of labor force instability from pandemics are not thoroughly explored in current evidence; hence further epidemiological surveillance is needed.

In conclusion, any change in labor force participation during COVID-19 was associated with elevated risk of depression in both sexes when compared to employed staying in a steady-state full-time employment. Becoming unemployed or working reduced employment hours were associated with the highest risk of depression as compared to employed individuals staying in a steady-state full-time employment.

## Supplementary data

Supplementary data are available at *EURPUB* online.

## Funding

This project was financed by a grant from the non-profit Velliv Foundation (grant number 20-0438). The Velliv Foundation did not have any influence on the analyses or interpretation of the results.

*Conflicts of interest*: The authors declare no conflicts of interest.

## Supplementary Material

ckac168_Supplementary_Data

## Data Availability

The data were processed in the data secure remote server environment of Statistic Denmark. The data underlying this article cannot be shared publicly due to ethical restrictions pertaining to Danish law for the privacy of individuals participating in the study. The data are available to authorized epidemiologists granted access by statistics Denmark to this project-specific data secure environment. Changes in labor force participation during coronavirus disease 2019 (COVID-19) elevated the risk of depression in both sexes. Risk of depression was pronounced in individuals with low labor force participation during COVID-19. Somatic comorbidity did not seem to modify this association. Knowledge on effects of changes in labor force participation on mental health is fundamental for early disease identification.
