# Supplementary material for: Labor force participation during COVID-19 and risk of depression: a Danish register study
Source: Eur J Public Health. 2022 Nov 18;33(1):80–6. doi: 10.1093/eurpub/ckac168 (PMC9897998; doi:10.1093/eurpub/ckac168)
Supplement: ckac168_Supplementary_Data [file ckac168_supplementary_data.zip › ckac168_Supplementary_Data/ejph-2022-06-om-0325-File002.docx]

Supplementary 1. Sex-stratified associations of long-term changes (February 2020 to August 2020) in labor force participation during COVID-19 with onset of depression in 3 113 308 Danes aged 25 to 67 years including clinical depression diagnoses from the primary and secondary healthcare sector in Denmark until 31 December 2020.

| Hazard risk (HR)  95% confidence interval (CI) | *Total (%)* | Cases, N | Person years, N | Incidence Rate | HR (CI)^a^ | HR (CI)^b^ |
| --- | --- | --- | --- | --- | --- | --- |
| ***Men*** | ***N=1 579 283 (100)*** | ***52 793*** | ***N=521 040*** | ***Per 1000 person years*** |  |  |
| Employment hours per week  No change  <30  30-36  ≥37  Increased employment hours  Decreased employment hours  Becoming employed  Becoming unemployed  Outside labor force^c^ | 89 500 (5.8)  69 755 (4.4)  671 524 (42.5)  107 750 (6.8)  147 415 (9.3)  51 042 (3.2)  62 125 (3.9)  380 172 (24.1) | 4 222  1 941  13 699  2 565  3 506  1 766  2 547  22 547 | 29 373  23 067  222 637  35 695  48 847  16 851  20 449  124 121 | 144  84  62  72  72  105  125  182 | 2.48 (2.39-2.56)  1.39 (1.33-1.46)  1.00  1.24 (1.18-1.29)  1.20 (1.16-1.25)  1.89 (1.80-1.99)  2.18 (2.09-2.28)  3.00 (2.94-3.07) | 2.20 (2.12-2.28)  1.35 (1.28-1.42)  1.00  1.23 (1.18-1.29)  1.22 (1.17-1.27)  1.76 (1.60-1.93)  2.11 (2.02-2.20)  2.59 (2.38-2.82) |
| ***Women*** | ***N=1 534 025 (100)*** | ***N=92 965*** | ***N=501 063*** |  |  |  |
| Employment hours per week  No change  <30  30-36  ≥37  Increased employment hours  Decreased employment hours  Becoming employed  Becoming unemployed  Outside labor force^c^ | 132 069 (8.6)  178 546 (11.6)  488 645 (31.9)  88 677 (5.8)  105 806 (6.9)  51 988 (3.4)  66 771 (4.4)  421 523 (27.4) | 10 385  9 209  16 647  3 788  4 591  3011  4 325  41 009 | 42 821  58 524  161 220  29 177  34 804  17 017  21 805  135 695 | 243  157  103  130  132  177  198  302 | 2.45 (2.39-2.51)  1.50 (1.46-1.54)  1.00  1.35 (1.30-1.40)  1.35 (1.31-1.39)  2.00 (1.93-2.08)  2.17(2.10-2.25)  3.10 (3.04-3.16) | 2.25 (2.20-2.31)  1.39 (1.35-1.43)  1.00  1.29 (1.24-1.34)  1.33 (1.28-1.37)  1.85 (1.72-2.00)  2.10 (2.02-2.16)  2.74 (2.56-2.93) |
| ^a^Age-adjusted.  ^b^Multivariate-adjusted: Age, comorbidity, ethnicity, residence of living, marital status, education, and industry.  ^c^Being outside labor force for various reasons: sickness leave, retirement, education, or unknown. | | | | | | |
