# Supplementary material for: Labor force participation during COVID-19 and risk of depression: a Danish register study
Source: Eur J Public Health. 2022 Nov 18;33(1):80–6. doi: 10.1093/eurpub/ckac168 (PMC9897998; doi:10.1093/eurpub/ckac168)
Supplement: ckac168_Supplementary_Data [file ckac168_supplementary_data.zip › ckac168_Supplementary_Data/ejph-2022-06-om-0325-File003.docx]

Supplementary 2. Associations of long-term changes (February 2020 to August 2020) in labor force participation during COVID-19 with onset of depression stratified by comorbidity in 3 113 308 aged 25 to 67 years including clinical diagnoses from the primary and secondary healthcare sector in Denmark until 31 December 2020.

| Charlson comorbidity index | Comorbidity | | | | | |
| --- | --- | --- | --- | --- | --- | --- |
|  | **Score 0** | | **Score 1-2** | | **Score 3+** | |
| Hazard risk (HR)  95% confidence interval (CI) | HR (CI)^a^ | HR (CI)^b^ | HR (CI)^a^ | HR (CI)^b^ | HR (CI)^a^ | HR (CI)^b^ |
| *N/E=Participants/events* | *N=2 709 059* |  | *N=349 587* |  | *N=54 662* |  |
|  | *E = 113 802* |  | *E = 26 418* |  | *E=5 538* |  |
| Employment hours per week  No change  <30  30-36  ≥37  Increased employment hours  Decreased employment hours  Becoming employed  Becoming unemployed  Outside labor force^c^ | 2.67 (2.61-2.73)  1.75 (1.71-1.79)  1.00  1.33 (1.29-1.37)  1.28 (1.25-1.32)  1.96 (1.90-2.03)  2.20 (2.14-2.27)  3.03 (2.99-3.08) | 2.24 (2.19-2.29)  1.36 (1.33-1.40)  1.00  1.27 (1.23-1.30)  1.28 (1.24-1.31)  1.89 (1.79-1.99)  1.74 (1.63-1.86)  2.58 (2.44-2.74) | 2.48 (2.36-2.61)  1.54 (1.45-1.63)  1.00  1.19 (1.10-1.29)  1.21 (1.13-1.30)  2.21 (2.03-2.39)  2.40 (2.25-2.57)  3.19 (3.07-3.30) | 2.18 (2.07-2.29)  1.28 (1.20-1.36)  1.00  1.17 (1.08-1.27)  1.22 (1.14-1.31)  2.24 (1.91-2.63)  2.28 (2.13-2.43)  3.10 (2.68-3.58) | 2.24 (1.96-2.55)  1.40 (1.16-1.69)  1.00  1.08 (0.84-1.39)  1.13 (0.91-1.40)  2.19 (1.76-2.72)  1.99 (1.67-2.38)  3.19 (2.90-3.52) | 2.08 (1.81-2.38)  1.19 (0.98-1.44)  1.00  1.12 (0.87-1.45)  1.17 (0.95-1.46)  1.99 (1.32-2.99)  1.96 (1.64-2.34)  2.77 (1.93-3.99) |
| ^a^Age-adjusted.  ^b^Multivariate-adjusted: Sex, age, ethnicity, residence of living, marital status, education, and industry.  ^c^Being outside labor force for various reasons: sickness leave, retirement, education, or unknown. | | | | | | |
