# Supplementary material for: Labor force participation during COVID-19 and risk of depression: a Danish register study
Source: Eur J Public Health. 2022 Nov 18;33(1):80–6. doi: 10.1093/eurpub/ckac168 (PMC9897998; doi:10.1093/eurpub/ckac168)
Supplement: ckac168_Supplementary_Data [file ckac168_supplementary_data.zip › ckac168_Supplementary_Data/ejph-2022-06-om-0325-File004.docx]

Supplementary 3. Multivariate-adjusted associations of long-term changes (February 2020 to August 2020) in labor force participation during COVID-19 with onset of mild, moderate, and severe depression in 3 113 308 Danes aged 25 to 67 years including in-hospital clinical depression diagnoses until 31 December 2020.

| Hazard risk (HR)  clinical depression | Mild  Events=1 126 | | Moderate  Events=4 636 | | Severe  Events=1 268 | |
| --- | --- | --- | --- | --- | --- | --- |
|  | HR^a^ | HR^b^ | HR^a^ | HR^b^ | HR^a^ | HR^b^ |
| Employment hours per week  No change  <30  30-36  ≥37  Increased employment hours  Decreased employment hours  Becoming employed  Becoming unemployed  Outside labor force**^c^** | 1.73 (1.31-2.28)  1.21 (0.89-1.66)  1.00  1.39 (1.00-1.92)  1.61 (1.21-2.14)  1.52 (0.79-2.91)  3.54 (2.71-4.62)  5.22 (4.41-6.19) | 1.76 (1.39-2.23)  1.33 (1.02-1.73)  1.00  1.43 (1.08-1.89)  1.56 (1.21-2.01)  1.74 (1.00-3.03)  3.59 (2.86-4.52)  3.55 (2.14-5.88) | 2.73 (2.41-3.10)  1.66 (1.44-1.92)  1.00  1.56 (1.34-1.82)  1.48 (1.28-1.71)  2.27 (1.91-2.70)  4.18 (3.67-4.76)  5.37 (4.94-5.84) | 2.25 (1.98-2.56)  1.36 (1.17-1.58)  1.00  1.48 (1.26-1.73)  1.45 (1.25-1.68)  2.20 (1.55-3.13)  3.73 (3.27-4.26)  4.87 (3.53-6.71) | 2.29 (1.81-2.91)  1.85 (1.45-2.36)  1.00  1.29 (0.95-1.74)  1.29 (0.98-1.69)  2.29 (1.68-3.13)  4.29 (3.41-5.41)  4.08 (3.50-4.76) | 1.93 (1.51-2.47)  1.62 (1.26-2.09)  1.00  1.27 (0.94-1.73)  1.31 (0.99-1.72)  1.35 (0.77-2.38)  3.77 (2.98-4.77)  2.18 (1.32-3.59) |
| ^a^Age-adjusted.  ^b^Multivariate-adjusted: Sex, age, comorbidity, ethnicity, residence of living, marital status, education, and industry.  ^c^Being outside labor force for various reasons: sickness leave, retirement, education, or unknown. | | | | | | |
